# A pseudo-analytic generalization of the memoryless property for continuous random variables and its use in pricing contingent claims

**DOI:** 10.1098/rsos.231690

**Published:** 2024-04-10

**Authors:** Peter Carr, Pasquale Cirillo

**Affiliations:** ^1^ Finance and Risk Engineering Department, New York University, New York, NY, USA; ^2^ ZHAW School of Management and Law, Institute of Business Information Technology, Winterthur, Switzerland

**Keywords:** memoryless property, pseudo-analysis, pseudo-sum, distribution theory, contingent claim

## Abstract

We explore an extension of the memoryless property for continuous random variables by using the concept of pseudo-sum. Subsequently, we demonstrate the practicality of this approach through two financial applications in which pseudo-sums characterize the values of arbitrage-free contingent claims. Moreover, we are able to establish new interesting connections between different probability distributions.

## 1. Introduction

Aczél characterizes all strictly increasing associative binary operations in abstract algebra, by relating them to the associativity functional equation [1,2]. In probability theory, one can characterize all memoryless continuous random variables, by relating their probability laws to the Cauchy exponential functional equation [3,4].

This article connects these two functional equations to each other. We relate Aczél’s characterization [1] of all associative and strictly increasing binary operations, as isomorphic to ordinary addition, to the well-known characterization of all continuous memoryless random variables as exponentially distributed. As a result, we obtain a generalization of the memoryless property in the continuous setting, which proves useful in financial applications and distribution theory. In particular, we discuss the case of two arbitrage-free contingent-claim values and the emerging relationships among some extreme value distributions.

Our work can be seen as a further application of the non-Newtonian approach developed by Grossman & Katz [5], and more recently extended by Pap [6] and co-authors. As we shall observe, our findings also share the rationale of some pioneering research by Kolmogorov [7] and de Finetti [8], among others.

This article is organized as follows. In §2, we define the generalized memoryless property (GMP). In §3, we present our view on how to characterize the GMP for a continuous random variable. In §4, we illustrate our findings with two examples, leading towards the financial applications of §5. In §6, exploiting the GMP, we provide new connections among notable distributions of the financial and actuarial literature, as well as of extreme value theory. Finally, §7 summarizes this article and suggests interesting future research. In the appendices, we collect the longer proofs and extra results, including some incidental findings that could be useful in the study of economic inequality.

## 2. A generalized memoryless property

A continuous random variable X shows the memoryless property [9] if, for all $x_{1},x_{2}\in R$ , one has:


(2.1) $$P\{X\geq x_{1}+x_{2}|X\geq x_{1}\}=P\{X\geq x_{2}\}.$$

This problem can be easily converted into a search for a strictly decreasing continuous solution S:R↦(0,1) , to the following Cauchy exponential functional equation [4]:


(2.2) $$S(a_{1}+a_{2})=S(a_{1})\times S(a_{2}),\;a_{1},a_{2}\in R.$$

It is well-known that the only strictly decreasing continuous solution to equation (2.2) with S(0)=1 is


(2.3) $$S(a)=e^{-\lambda a},\;a\geq 0,$$

for some positive constant λ [1].

In other words, the only continuous law satisfying the memoryless property of equation (2.1) is the exponential distribution, whose survival function (SF) is actually given in equation (2.3).

However, the Cauchy exponential functional equation in equation (2.2) can also be expressed in a different manner. Let p=S(a) , where the function S:R↦(0,1) is still strictly decreasing and continuous. Then, there exists a strictly decreasing and continuous inverse function $S^{-1}:(0,1)\mapsto R$ , such that $a=S^{-1}(p)$ . By substituting $a_{1}=S^{-1}(p_{1})$ and $a_{2}=S^{-1}(p_{2})$ into equation (2.2), one gets


(2.4) $$S\left(S^{-1}(p_{1})+S^{-1}(p_{2})\right)=p_{1}\times p_{2},\;p_{1},p_{2}\in (0,1].$$

Hence, addition + between non-negative reals $a_{i}\in [0,\infty )$ , with i=1,2 , is *isomorphic* to multiplication × between probabilities $p_{i}\in (0,1]$ .

The strictly decreasing continuous function *S*, generating the multiplication monoid ((0,1]:×,1) from the addition monoid ([0,∞):+,0) , is the strictly decreasing exponential function given in equation (2.3). This function *S* is called the *generator* of the multiplication monoid [10].

The generator of a monoid surely needs to be strictly monotonic, but it does not necessarily need to be exponential. By changing the generator *S*, one can, in fact, change the binary operation that is isomorphic to ordinary addition. Our GMP simply replaces the ordinary sum $x_{1}+x_{2}$ on the left-hand side of equation (2.1) with the so-called pseudo-sum $x_{1}⊕x_{2}$ .

Following the pioneering work of Grossman & Katz [5], further elaborated by Pap [6], a pseudo-sum is the result of applying the binary operation ⊕ in a monoid (A:⊕,e) , where A is now a connected subset [ℓ,u] of the extended real line [−∞,∞] . As a result, we have the following properties:

- *closure*: for $x_{1},x_{2}\in A$ , then $x_{1}⊕x_{2}\in A$ ;
- *existence of an identity element*: there exists e∈A , such that x⊕e=e⊕x=x , for all x∈A ; and
- *associativity*: for $x_{1},x_{2},x_{3}\in A$ , one has that $\left(x_{1}⊕x_{2})⊕x_{3}=x_{1}⊕(x_{2}⊕x_{3}\right)$ .

In the general setting of [5] and [6], the monoid (A:⊕,e) is not required to be commutative, or cancellative, or to have an inverse element in the set.

In this article, the set A=[ℓ,h] over which the monoid of interest is defined will be the support of the law of a continuous^1^ random variable *X*.

When the associativity requirement of a monoid is dropped, the resulting algebraic structure is called an *unital magma* [10]. Let us thus consider an unital magma $\left(S:{\hat{⊕}}_{G},e\right)$ where A=[ℓ,h] is a connected subset of the extended real line [−∞,∞] , possibly [−∞,∞] itself, and whose binary operation ${\hat{⊕}}_{G}$ is strictly increasing but not necessarily associative. It turns out that, in this context, additionally demanding the associativity of the binary operation ${\hat{⊕}}_{G}$ puts a severe constraint on its form.

Let ${⊕}_{G}$ denote the binary operation in the unital magma $\left(A:{⊕}_{G},e\right)$ , when it is required to be both strictly increasing and associative. Then, $\left(A:{⊕}_{G},e\right)$ is also a monoid, and there must exist a strictly monotonic function G:R↦A such that [2]:


(2.5) $$x_{1}{⊕}_{G}x_{2}=G(G^{-1}(x_{1})+G^{-1}(x_{2})),$$

where $G^{-1}:A\mapsto R$ denotes the inverse of the generator *G*.

Therefore,^2^ requiring that the strictly increasing binary operation ${\hat{⊕}}_{G}$ in the magma $\left(A:{\hat{⊕}}_{G},e\right)$ also be associative forces the resulting binary operation ${⊕}_{G}$ to be isomorphic to ordinary addition + over the reals, where the isomorphism is given by equation (2.5). The strictly monotonic function G:R↦A in equation (2.5) is called the generator of the strictly increasing binary operation ${⊕}_{G}$ in the monoid $\left(A:{⊕}_{G},e\right)$ . It turns out that this monoid must be commutative, cancellative, and that, for the identity element, e=G(0) .

**Definition 2.1** (GMP). The law of a continuous random variable X , with support A=[ℓ,h] , is said to enjoy the GMP if, for all $x_{1},x_{2}\in A$ , the probability measure P satisfies either


(2.6) $$P\{X\geq x_{1}{⊕}_{G}x_{2}|X\geq x_{1}\}=P\{X\geq x_{2}\},$$

or


(2.7) $$P\{X\leq x_{1}{⊕}_{G}x_{2}|X\leq x_{1}\}=P\{X\leq x_{2}\},$$

where ${⊕}_{G}$ indicates a pseudo-sum, which is strictly increasing in both $x_{1}\in A$ and $x_{2}\in A$ .

From now on, we restrict the domain of the strictly monotonic generator G to the non-negative real line [0,∞) . Moreover, we demand that the strictly monotonic generator *G* maps all of [0,∞) onto the support A=[ℓ,u] of the continuous random variable *X* . Since the generator *G* is a strictly monotonic map from all of [0,∞) onto A , its inverse map $G^{-1}$ must also be a strictly monotonic map from all of A onto [0,∞) .

As a consequence, we have that:

- if G is strictly increasing, then so is $G^{-1}$ , and the continuous random variable X with support A=[ℓ,u] enjoys the GMP as per equation (2.6); and
- if G is strictly decreasing, then so is $G^{-1}$ , and the continuous random variable X with support A=[ℓ,u] enjoys the GMP as per equation (2.7).

All in all, the law of a continuous random variable X supported on A=[ℓ,h] enjoys the GMP, with + replaced by the strictly increasing associative binary operation ${⊕}_{G}$ , if and only if the strictly monotonic generator G of ${⊕}_{G}$ maps all of [0,∞) onto the support A=[ℓ,h] of X . Hence, G is a bijection between A and [0,∞) .

This restriction on the type of monotonicity of G , the domain of G and the image of G is one of the contributions of this article. Together with the probabilistic and financial interpretations of the pseudo-sums involved in the GMP, it distinguishes our work from similar intuitions in references [3,11].

The reader might have noticed that the GMP naturally connects to other useful concepts of probability and statistics, like, for example, the generalized means of Kolmogorov, Nagumo, de Finetti & Chisini [7,8,12]. As observed in [5], thanks to different appropriate choices of the generator G , the operation ${⊕}_{G}$ can define ‘alternative universes’, in which addition is, for instance, replaced by multiplication, the natural integral is the geometric integral, and the familiar arithmetic mean is somehow replaced by the geometric one. In such a multiplicative universe, the random variable showing the memoryless property would not be the exponential but rather the Pareto (see §4), and the random variable playing the role of the normal in many useful convergence results could be the lognormal (see §7 for an intuition). A universe like that would therefore be a natural place to study phenomena of growth, contagion, cascading failures and so on [5]. Moving across all these ‘universes’ thus often provides ways to simplify or extend known facts, discovering interesting relations and applications, as we try to do in the next sections.

## 3. Bijections between [0,∞) and A that allow for the generalized memoryless property

Again, let X be a continuous random variable supported on a connected interval A=[ℓ,u] , which is a subset of the extended real line [-∞,∞] . We allow ℓ=-∞ and/or u=∞ .

The SF of X is a strictly decreasing map $S_{X}\,\left(x\right)$ from A to the unit interval, [0,1] . We require that $S_{X}\,\left(\ell \right)=1$ and that $S_{X}\,\left(u\right)=0$ .

Let I be a strictly increasing function I:[ℓ,u]↦[0,∞) with I⁢(ℓ)=0 and I(u)=∞ . We call the function I(x) the *Laplace exponent* in the Laplace representation of $S_{X}\,\left(x\right)$ if


(3.1) $$S_{X}(x)=e^{-\lambda I(x)},\;x\in [\ell ,u],$$

where λ>0 is a positive constant called the Laplace parameter [2].

The cumulative distribution function (CDF) of X , denoted by $F_{X}\,\left(x\right)$ , is defined by


(3.2) $$F_{X}(x)\equiv 1-S_{X}(x),\;x\in [\ell ,u].$$

Obviously, the CDF of X is a strictly increasing map from the support A of X to the unit interval [0,1] , with $F_{X}\,\left(\ell \right)=0$ and $F_{X}\,\left(u\right)=1$ .

Let D⁢(x) be a strictly decreasing function D:[ℓ,u]↦[0,∞) with D⁢(ℓ)=∞ and D⁢(u)=0 . We call the function D⁢(x) the Laplace exponent in the Laplace representation of $F_{X}(x)$ if


(3.3) $$F_{X}(x)=e^{-\lambda D(x)},\;x\in [\ell ,u],$$

where again λ>0 is a positive constant.

Since I⁢(⋅) is a strictly increasing map of all of A onto [0,∞) , its inverse $I^{-1}(\cdot )$ is a strictly increasing map of all of [0,∞) onto A . Likewise, since D⁢(⋅) is a strictly decreasing map of all of A onto [0,∞) , its inverse $D^{-1}\,\left(\cdot \right)$ is a strictly decreasing map of all of [0,∞) onto A .

Let a denote an arbitrary element in the additive monoid ([0,∞):+,0) . Suppose that a monotonic generator G⁢(a) produces an isomorphic monoid (A:⊕,e) , where A is as usual a connected open subset of ℝ , ⊕ is the monoid’s binary operation and e is the monoid’s identity element. If the generator G(a) is strictly monotonic, then we denote the binary operation by ${⊕}_{G}$ and the identity element by G⁢(0) . From equation (2.5), the binary operation ${⊕}_{G}$ in the isomorphic monoid $\left(A:{⊕}_{G},G(0)\right)$ is strictly increasing.

We henceforth focus our attention on the strict subset of the class of all strictly monotonic generators which enforce a bijective map between [0,∞) and A , which is the support of the random variable X . We call such generators *feasible* for X .

If the X- feasible generator G is strictly increasing, then we set $G(a)=I^{-1}(a)$ , so that $I(x)=G^{-1}(x)$ is a strictly increasing map of all of [ℓ,u] onto [0,∞) . If the X- feasible generator G is instead strictly decreasing, then we set $G\,\left(x\right)=D^{-1}\,\left(x\right)$ so that $D(x)=G^{-1}(x)$ is a strictly decreasing map of all of [ℓ,u] onto [0,∞) .

When we obtain the Laplace exponent I⁢(x) (or D⁢(x) ) by inverting an X- feasible generator G , then the SF (or the CDF) defined by equation (3.1) (or equation (3.3)) is guaranteed to strictly decrease from 1 to 0 (or to strictly increase from 0 to 1) as required.

If the X- feasible generator G⁢(a) is strictly increasing, then the SF generated by equation (3.1) satisfies the functional equation


(3.4) $$S_{X}\,\left(x_{1}{⊕}_{G}x_{2}\right)=S_{X}\,\left(x_{1}\right)\times S_{X}\,\left(x_{2}\right),$$

which leads to the GMP in equation (2.6), that is, once again,


(3.5) $$\mathbb{P}\,\left\{X\geq x_{1}{⊕}_{G}x_{2}|X\geq x_{1}\right\}=\mathbb{P}\,\left\{X\geq x_{2}\right\},$$

where *x*_1_, *x*_2_ and $x_{1}{⊕}_{G}x_{2}$ are all in the support A=[ℓ,u] of X .

If the X- feasible generator G⁢(a) is instead strictly decreasing, then the CDF generated by equation (3.3) satisfies the functional equation


(3.6) $$F_{X}\,\left(x_{1}{⊕}_{G}x_{2}\right)=F_{X}\,\left(x_{1}\right)\times F_{X}\,\left(x_{2}\right),$$

which leads to the GMP in equation (2.7), restated here as


(3.7) $$\mathbb{P}\,\left\{X\leq x_{1}{⊕}_{G}x_{2}|X\leq x_{1}\right\}=\mathbb{P}\,\left\{X\leq x_{2}\right\},$$

where again *x*_1_, *x*_2_ and $x_{1}{⊕}_{G}x_{2}$ are all in the support A=[ℓ,u] of X .

To illustrate these results, let us first consider the identity map for the generator G , that is, G⁢(a)=a for a≥0 .

Since this generator is a strictly increasing map from all of [0,∞) to [0,∞) , it follows that G⁢(a)=a is feasible for a continuous random variable X supported on [0,∞) . The inverse of G is clearly an increasing map, so we set I⁢(x)=x in equation (3.1) for the SF of X . The continuous random variable X is thereby identified as being exponentially distributed with parameter λ>0 .

Trivially, when G⁢(a)=a , one gets that ${⊕}_{G},=,+$ , and the GMP reduces to the standard memoryless property of the exponential random variable [9], as per equation (2.1).

## 4. Examples of bijective generators

Let us now take into consideration some examples of random variables manifesting the GMP. Of two of them, we will discuss the financial implications in §5, to show their applicability in financial engineering.

### 4.1. The generator G is an increasing power function

A first basic example of a strictly increasing generator is the power function


(4.1) $$G(a)=a^{b},\;a\geq 0,b>0.$$

Since this generator maps all of [0,∞) onto [0,∞) , it is feasible for a continuous random variable X supported on [0,∞) .

Letting $x\equiv a^{b}\geq 0$ , the inverse of the generator G defined in equation (4.1) is


(4.2) $$G^{-1}(x)=x^{\frac{1}{b}}\equiv I(x),\;x\geq 0,b>0.$$

This inverse is also strictly increasing.

Setting $I\,\left(x\right)=x^{\frac{1}{b}}$ in equation (3.1), the resulting SF is that of a Weibull [13], that is:


(4.3) $$S_{X}(x;\lambda ,b)=e^{-\lambda x^{\frac{1}{b}}},\;x\in [0,\infty ),\lambda >0,b>0.$$

Setting $G^{-1}\,\left(x\right)=I\,\left(x\right)=x^{\frac{1}{b}}$ in equation (2.5), the corresponding binary operation ${⊕}^{b}$ is an $\ell^{p}$ norm of the vector $\left[x_{1},x_{2}\right]$ , with p=1/b :


(4.4) $$x_{1}{⊕}^{b}x_{2}\equiv {\left(x_{1}^{\frac{1}{b}}+x_{2}^{\frac{1}{b}}\right)}^{b},\;x_{1}\in [0,\infty ),x_{2}\in [0,\infty ),b>0.$$

For $x_{1},x_{2}\in \left[0,\infty \right)$ , and b>0 , the following GMP holds for the Weibull-distributed random variable X≥0 , whose SF is in equation (4.3):


(4.5) $$\mathbb{P}\,\left\{X\geq x_{1}{⊕}^{b}x_{2}|X\geq x_{1}\right\}=\mathbb{P}\,\left\{X\geq x_{2}\right\}.$$

It is worth noticing that equation (4.5) recalls the functional equation that Wang [14] provides to characterize Weibull-distributed random variables.

### 4.2. The generator G is a negated logarithm

Suppose now that the generator is the following negated (natural) logarithm:


(4.6) G(a)=−blog⁡a,a>0,b>0.

The right-hand side of equation (4.6) can be written as the negative of a logarithm whose base is $e^{\frac{1}{b}}$ . As a result, we treat the parameter b>0 in equation (4.6) as the base controller.

Note that the generator defined by equation (4.6) is strictly decreasing.

Since this G maps all of [0,∞) to (-∞,∞] , the generator G defined in equation (4.6) is feasible for a continuous random variable X supported on (-∞,∞] .

Letting x≡-b⁢log⁡a , for a≥0,b>0 , we observe that x∈(-∞,∞] . As a result, the inverse of the generator G defined in equation (4.6) is


(4.7) $$G^{-1}(x)=e^{-\frac{x}{b}}\equiv D(x),\;x\in (-\infty ,\infty ],b>0.$$

This inverse is also strictly decreasing.

Setting $D\,\left(x\right)=e^{-\frac{x}{b}}$ , with x∈(-∞,∞] and b>0 , in (3.3), the resulting CDF is Gumbel [13], that is:


(4.8) $$F_{X}(x;\lambda ,b)=e^{-\lambda e^{-\frac{x}{b}}},\;x\in (-\infty ,\infty ],\lambda >0,b>0.$$

Setting $G^{-1}\,\left(x\right)=D\,\left(x\right)=e^{-\frac{x}{b}}$ in equation (2.5), the corresponding binary operation, ${⊕}_{-b}$ , is the following log-sum-exponential:


(4.9) $$x_{1}{⊕}_{-b}x_{2}=-b\log{\left(e^{-\frac{x_{1}}{b}}+e^{-\frac{x_{2}}{b}}\right)}^{b},\;x_{1}\in (-\infty ,\infty ],x_{2}\in (-\infty ,\infty ],b>0.$$

Note that this pseudo-sum is itself increasing in $x_{1},x_{2}\in \left(-\infty ,\infty \right]$ .

For b>0 , the following GMP holds for the Gumbel-distributed random variable X∈(-∞,∞] , whose CDF is given in equation (4.8):


(4.10) $$P\{X\leq x_{1}{⊕}_{-b}x_{2}|X\leq x_{1}\}=P\{X\leq x_{2}\}.$$

### 4.3. Other possibilities

Many examples of the GMP emerge if one chooses different generators. For example, when $G(a)=e^{a}$ , a≥0 , one has $x_{1}{⊕}_{G}x_{2}=x_{1}\times x_{2}$ , and the random variable showing the GMP is a standard Pareto. Interestingly, as observed by Chisini [12] and de Finetti [8], when speaking about means, this is also the situation in which the arithmetic mean we are all familiar with is naturally replaced by the geometric mean as the reference mean. This comes from the fact that + is replaced by × , thanks to *G*.

Simple generalizations can be obtained by adding, for instance, a constant c>0 , so that G(a)=exp⁡(a+c) . These generalizations all lead to different Paretian random variables, like type II and type III [13].

Alternatively, choosing $G(a)=\log e^{a}-1$ , with a>0 , generates the pseudo-sum $x_{1}{⊕}_{G}x_{2}=\log\left(e^{x_{1}+x_{2}}+e^{x_{1}}+e^{x_{2}}\right)$ , and the GMP belongs to a standard logistic random variable. Interestingly, in this case, $G^{-1}(x)=\log e^{x}+1$ , which is nothing more than the integrated CDF of the same logistic random variable [13], linked to the well-known softplus activation function in machine learning [15,16].

Many other examples can naturally be thought of, but our goal here is not to provide a complete taxonomy of all possible cases of GMP, as it would be unrealistic. We rather prefer to show the usefulness of this alternative way of approaching memorylessness via pseudo-sum, when considering some financial and probabilistic applications. For this reason, in the rest of this article, we focus our attention on the two generators of §4.1 and §4.2.

## 5. Financial interpretations of ${⊕}^{b}$ and ${⊕}_{-b}$

The pseudo-sums $x_{1}{⊕}^{b}x_{2}$ and $x_{1}{⊕}_{-b}x_{2}$ , as defined in equations (4.4) and (4.9) and 4_9 of §4, can be both interpreted as the arbitrage-free values of particular contingent claims. This strengthens the idea of the usefulness of non-Newtonian calculus in finance, as already observed in [17,18].

As a matter of fact, pseudo-sums seem to emerge naturally in many relevant aspects of financial mathematics. For instance, one could already notice that, in the basic Black–Scholes–Merton setting (BSM) [19], when prices follow a geometric Brownian motion, the change in measure between ℙ (market or physical measure) and ℚ (risk-neutral measure), controlled by the so-called Wang transform [20] is just example of a pseudo-sum.

Let S(t)≥0 be the price of a given asset at time t∈[0,T] under the physical measure ℙ . Let K>0 be a strike price. Fix a value S(0)≥0 and assume that


$$S\,\left(t\right)=S\,\left(0\right)\,\exp\left(\left(\mu -\frac{\sigma^{2}}{2}\right)\,t+\sigma \,B\,\left(t\right)\right),$$

where μ∈ℝ , σ>0 and B⁢(t) is a standard Brownian motion under ℙ .

Hence, we have that, in T , under ℙ :


$$\log S\,\left(T\right)∼\Phi \,\left(\log S\,\left(0\right)+\left(\mu -\frac{1}{2}\,\sigma^{2}\right)\,T,\sigma \,\sqrt{T}\right),$$

where Φ⁢(⋅) is the CDF of a standard normal.

If we introduce Q as the risk-neutral measure equivalent to P , we also know [19] that, under Q,


$$\log S(T)∼\Phi \left(\log S(0)+\left(r-\frac{1}{2}\sigma^{2}\right)T,\sigma \sqrt{T}\right),$$

where r≥0 is the so-called risk-free rate.

The connection between ℙ and ℚ in the basic BSM framework has been studied explicitly in [20], showing that


(5.1) $$Q(S_{T})=Q(\log S_{T}>\log K)=\Phi \left(\Phi^{-1}(\underset{p}{\underbrace{P(\log S_{T}>\log K)}})-\frac{(\mu -r)\sqrt{T}}{\sigma }\right),$$

where $\frac{\left(\mu -r\right)}{\sigma }$ is clearly the Sharpe ratio [19], and $\Phi^{-1}\,\left(\cdot \right)$ is the quantile function associated with Φ⁢(⋅) . Equation (5.1) thus shows that Q is a distortion of P , obtained via a quantile shift, which induces a re-weighting of the probabilities.

Simple manipulations also bring to


(5.2) $$P(S_{T}>K)=P(\log S_{T}>\log K)=\Phi \left(\Phi^{-1}(Q(\log S_{T}>\log K))+\frac{(\mu -r)\sqrt{T}}{\sigma }\right).$$

Equation (5.1) is an example of application of the function


(5.3) $$\Phi \left(\Phi^{-1}(p)-c\right),$$

known as Wang transform [20], which represents a useful distortion function in the actuarial literature. Such a function is convex for c>0 and concave for c<0 . When c=0 , no distortion clearly takes place.

Now, it is not difficult to observe that the Wang transform is just a special case of pseudo-sum—or, if one prefers, of pseudo-difference—in fact


(5.4) $$\Phi \,\left(\Phi^{-1}\,\left(p\right)-c\right)=\Phi \,\left(\Phi^{-1}\,\left(p\right)-\Phi^{-1}\,\left(\Phi \,\left(c\right)\right)\right).$$

This is just the first simple example. In fact, one could also play with models for which the Wang transform is substituted by the more general


(5.5) $$F\left(F^{-1}(p)-F^{-1}\left(F\left(c\right)\right)\right)=F\left(F^{-1}(p)-c\right),$$

where F is the CDF of a given unimodal random variable linked to the log returns of an asset of interest. Incidentally, both equations (5.3) and (5.5) and 5_5 also represent two proper Lorenz functions of the socioeconomic inequality literature [13,21], whose application in risk management has been investigated in [22], among others.

Further examples could be given, for example, by introducing new derivatives like Stoptions [17]. However, let us now come back to our pseudo-sums ${⊕}^{b}$ and ${⊕}_{-b}$ and to their financial interpretations, in line with the price generator views of [23].

### 5.1. The pseudo-sum ${⊕}^{b}$ and a zero-coupon default-free convertible bond

When the induced binary operation is ${⊕}^{b}$ , then for $x_{1},x_{2}\in \left[0,\infty \right)$ , and b∈(0,1) , one can observe that


(5.6) $$x_{1}{⊕}^{b}x_{2}\equiv {\left(x_{1}^{\frac{1}{b}}+x_{2}^{\frac{1}{b}}\right)}^{b}=E(x_{1}\vee x_{2}D_{b}),$$

where $D_{b}$ is a mean-one conjugate power Dagum (CPD) distributed random variable (see appendix A for more details) with CDF:


(5.7) $$P\{D_{b}\leq d\}={\left(1+d^{-\frac{1}{b}}\right)}^{b-1},\;d>0.$$

The right-hand side of equation (5.6) is nothing but the arbitrage-free value of a zero-coupon default-free convertible bond with face value $x_{1}\geq 0$ and conversion value $x_{2}\geq 0$ . The positive random variable $D_{b}>0$ is the gross return on the convertible bond’s underlying stock. Incidentally, notice that $D_{b}$ also defines a Radon–Nikodym derivative [19], and this further justifies the use of CPD random variables, as, for instance, in [24].

To prove that equation (5.6) holds, notice that its right-hand side can be written as


(5.8) $$\begin{array}{ll} E[x_{1}\vee (x_{2}D_{b})] & =E\{x_{2}D_{b}+[(x_{1}-x_{2}D_{b})\vee 0]\}\\ & =x_{2}E[D_{b}]+E[(x_{1}-x_{2}D_{b})\vee 0]\\ & =x_{2}+x_{2}E\left[\left(\frac{x_{1}}{x_{2}}-D_{b}\right)\vee 0\right] \end{array},$$

since $x_{2}>0$ , and $E[D_{b}]=1$ , as per appendix A.

Now observe that the multiplier of *x*_2_ is the value of a put written on $D_{b}$ and struck at $\frac{x_{1}}{x_{2}}$ . This put value is the integral of the distribution function of the mean-one CPD random variable, that is:


(5.9) $$\begin{array}{ll} E[x_{1}\vee (x_{2}D_{b})] & =x_{2}+x_{2}\int_{\;\;0}^{\frac{x_{1}}{x_{2}}}F_{D_{b}}(d)\text{d}gd\\ & =x_{2}+x_{2}\int_{\;\;0}^{\frac{x_{1}}{x_{2}}}{\left(1+d^{-\frac{1}{b}}\right)}^{b-1}\text{d}d\\ & =x_{2}+x_{2}{\left(1+d^{\frac{1}{b}}\right)}^{b}{|}_{d=0}^{d=\frac{x_{1}}{x_{2}}}\\ & =x_{2}+x_{2}\left[{\left(1+{\left(\frac{x_{1}}{x_{2}}\right)}^{\frac{1}{b}}\right)}^{b}-1\right]\\ & =x_{2}+{\left(x_{2}^{\frac{1}{b}}+x_{1}^{\frac{1}{b}}\right)}^{b}-x_{2}\\ & ={\left(x_{1}^{\frac{1}{b}}+x_{2}^{\frac{1}{b}}\right)}^{b} \end{array}.$$

This proves that equation (5.6) holds.

### 5.2. The pseudo-sum ${⊕}_{-b}$ and a zero-coupon defaultable bond

When the induced binary operation is ${⊕}_{-b}$ , then for $x_{1},x_{2}\in \left(-\infty ,\infty \right]$ , and b>0 , one can notice that


(5.10) $$x_{1}{⊕}_{-b}x_{2}=-b\log{\left(e^{-\frac{x_{1}}{b}}+e^{-\frac{x_{2}}{b}}\right)}^{b}=E[x_{1}\wedge (x_{2}+bZ)],$$

where Z is a standard logistic random variable with a distribution function [13]:


(5.11) $$\mathbb{P}\,\left\{Z\leq z\right\}={\left(1+e^{-z}\right)}^{-1},z\in \mathbb{R}.$$

The right-hand side of equation (5.10) is now the arbitrage-free value of a zero-coupon defaultable bond with face value $x_{1}\in \left(-\infty ,\infty \right]$ and whose underlying security has initial value $x_{2}\in \left(-\infty ,\infty \right]$ . The real-valued random variable b⁢Z is the change in the value of the operations of the firm.

To prove that equation (5.10) holds, notice that its right-hand side can be written as


(5.12) $$\begin{array}{ll} E[x_{1}\wedge (x_{2}+bZ)] & =x_{1}+E[0\wedge (x_{2}-x_{1}+bZ)]\\ & =x_{1}-\{-E[0\wedge (x_{2}-x_{1}+bZ)]\}\\ & =x_{1}-E[0\vee (x_{1}-x_{2}-bZ)]\\ & =x_{1}-bE\left[0\vee \left(\frac{x_{1}-x_{2}}{b}-Z\right)\right] \end{array}.$$

The multiplier of b is the value of a put written on Z and struck at $\frac{x_{1}-x_{2}}{b}$ . This put value is the integral of the standard logistic distribution function, that is:


(5.13) $$\begin{array}{ll} E[x_{1}\wedge (x_{2}+bZ)] & =x_{1}-b\int_{-\infty }^{\frac{x_{1}-x_{2}}{b}}F_{Z}(z)\text{d}z\\ & =x_{1}-b\int_{-\infty }^{\frac{x_{1}-x_{2}}{b}}{\left(1+e^{-z}\right)}^{-1}\text{d}z\\ & =x_{1}-b\log\left(1+e^{z}\right){|}_{z=-\infty }^{z=\frac{x_{1}-x_{2}}{b}}\\ & =-b\log\left(e^{-\frac{x_{1}}{b}}\right)-b\log\left(1+e^{\frac{x_{1}-x_{2}}{b}}\right)\\ & =-b\log\left[\left(e^{-\frac{x_{1}}{b}}\right)\times \left(1+e^{\frac{x_{1}-x_{2}}{b}}\right)\right]\\ & =-b\log\left(e^{-\frac{x_{1}}{b}}+e^{\frac{-x_{2}}{b}}\right) \end{array}.$$

This proves that equation (5.10) holds.

## 6. New connections between notable distributions

Exploiting the pseudo-sums of §4 and the relative GMP, we now offer new ways of connecting random variables largely used in the financial, actuarial and economic inequality literature [13]. These new links may open to better characterizations, as well as to more reliable modelling of important economic quantities. Interestingly, the random variables under scrutiny are all recurrent objects in extreme value theory [25], suggesting the existence of some non-trivial research paths for the future, as we sketch in §7.

In appendices B and C, the interested reader can find proofs and additional results.

### 6.1. New relationship between Weibull and Dagum distributions

Suppose that a positive random variable X>0 is exponentially distributed with SF $e^{-\lambda x}$ , x>0 , where λ>0 is a positive intensity parameter. For p>0 , let


(6.1) $$W=X^{\frac{1}{p}}$$

be a positive power of this exponential random variable. It is well-known that the distribution of W is Weibull with SF $e^{-{\left(\theta \,w\right)}^{p}}$ , w>0 , where $\theta =\lambda^{\frac{1}{p}}$ is the rate parameter [13,14].

Now let $K=\frac{1}{W}$ . Then, the distribution of K is called inverse Weibull with CDF equal to


(6.2) $$F_{K}(k;\theta ,p)=e^{-{\left(\frac{\theta }{k}\right)}^{p}},\;k>0,\theta >0,p>0.$$

The parameter θ is now a scale parameter. Let us randomize this scale parameter using an independent positive random variable.

Suppose that an independent positive random variable Y>0 is gamma-distributed with scale parameter σ>0 and shape parameter α>0 . The CDF of Y is $F_{Y}(y;\sigma ,\alpha )=\Gamma \left(\frac{y}{\sigma };\alpha \right)$ , where


(6.3) $$\Gamma \left(u;\alpha \right)\equiv \int_{\;\;0}^{u}t^{\alpha -1}e^{-t}\text{d}t,\;u>0,\alpha >0,$$

is the incomplete gamma function. For p>0 , let $\Theta =Y^{\frac{1}{p}}$ be a positive power of this gamma-distributed random variable. Note that we are using the same power $\frac{1}{p}>0$ that was used in equation (6.1) to transform the exponentially distributed random variable X into the Weibull-distributed random variable W .

The random variable $\Theta =Y^{\frac{1}{p}}$ is said to be a power-transformed gamma (PTG) with CDF:


(6.4) $$F_{\Theta }\,\left(\theta ;S,\alpha ,p\right)=\Gamma \,\left({\left(\frac{\theta }{S}\right)}^{p};\alpha \right),$$

where $S\equiv \sigma^{\frac{1}{p}}$ is the scale parameter. Note that the PTG distribution has three positive parameters that we call: scale S>0 , power p>0 and shape α>0 .

Suppose that one randomizes out the scale θ>0 of the inverse Weibull-distributed random variable K>0 using the independent and just-introduced PTG-distributed random variable Θ>0 . Let D denote the resulting positive random variable, whose law is determined by the three positive parameters of the PTG distribution. Then, appendix C shows that the CDF of D is


(6.5) $$F_{D}(d;S,p,\alpha )={\left(1+{\left(\frac{d}{S}\right)}^{-p}\right)}^{-\alpha },\;d>0,S>0,p>0,\alpha >0.$$

This CDF is that of a Dagum-distributed random variable, often used in economics and insurance studies, for example, to model income, wealth and actuarial losses [13].

While the positive parameter S>0 controls the scale of D>0 , it is not the mean of D . The mean of D is in fact infinite for p∈(0,1] . To obtain a positive random variable with a finite mean, we henceforth restrict our attention to the case p>1 .

It follows that


(6.6) $$b\equiv \frac{1}{p}$$

is bounded between 0 and 1. Suppose that we restrict the positive shape parameter α§gt;0 in equation (6.5) by forcing


(6.7) α=1−b.

With p>1 and hence b∈(0,1) , the restriction on α in equation (6.7) keeps it positive.

Let $D_{S,b}$ denote the random variable resulting from D by imposing equations (6.6) and (6.7) and 6_7. The two subscripts on D indicate the two positive parameters of this restricted Dagum law, with S>0 controlling the scale of $D_{S,b}>0$ , and with b∈(0,1) controlling its shape.

To name this restricted Dagum random variable, notice that, if we re-parametrize the three-parameter Dagum CDF in equation (6.5) by replacing α>0 with $q\equiv \frac{1}{\alpha }>0$ , then it becomes


(6.8) $$F_{D}(d;S,p,q)\equiv {\left(1+{\left(\frac{d}{S}\right)}^{-p}\right)}^{-\frac{1}{q}},\;d>0,S>0,p>0,q>0.$$

Under this representation, the restriction in equation (6.7) becomes $\alpha =\frac{1}{q}=1-b=1-\frac{1}{p}$ from equation (6.6). Since we have imposed the conjugate power restriction $\frac{1}{q}+\frac{1}{p}=1$ on equation (6.8) to eliminate a parameter, we refer to the resulting two-parameter law as CPD and to $D_{S,b}$ as a CPD-distributed random variable. Interestingly, the CPD distributions have been recently used in [24] for compound option pricing.

Setting $p=\frac{1}{b}$ and q=1−b in equation (6.8), the CDF of $D_{S,b}$ is


(6.9) $$F_{D_{S,b}}(d;S,b)\equiv {\left(1+{\left(\frac{d}{S}\right)}^{-\frac{1}{b}}\right)}^{b-1},\;K>0,S>0,b\in (0,1).$$

Appendix A proves that the mean of the positive CPD-distributed random variable $D_{S,b}$ is just its positive scale parameter S>0 . When the scale parameter S=1 , we refer to the mean-one positive random variable $D_{1,b}$ by simply $D_{b}$ .

Setting S=1 in equation (6.9) causes the CDF of $D_{b}$ to simplify into the one given in equation (5.7). Always in appendix A, we also provide the Lorenz curve and the Gini index associated with the distribution of the $D_{b}$ random variable, given its possible use as a statistical size distribution [13].

By connecting results from the last section with this one, we can finally introduce a new relationship between Dagum- and Weibull-distributed random variables.

When a random variable X is Weibull-distributed with SF given in equation (4.3), then we have from the GMP of equation (4.5) that


(6.10) $$\mathbb{P}\,\left\{X\geq x_{1}{⊕}^{b}x_{2}|X\geq x_{1}\right\}=\mathbb{P}\,\left\{X\geq x_{2}\right\}.$$

However, from equation (5.10), we know that for $x_{1},x_{2}\in \left[0,\infty \right)$ and b∈(0,1) , one has


(6.11) $$x_{1}{⊕}^{b}x_{2}=E(x_{1}\vee x_{2}D_{b}),$$

with $D_{b}$ being a mean-one CPD-distributed random variable, whose CDF is given in equation (5.7) .

Therefore, when the parameters p and q in the Dagum CDF equation (6.8) are conjugate, that is,


(6.12) $$\frac{1}{p}+\frac{1}{q}=1,\;p>1,q>1,$$

and we define $b=\frac{1}{p}\in \left(0,1\right)$ , then this special case of the Dagum distribution called CPD is connected to the Weibull distribution via equations (6.10) and (6.11) and 6_11, rather than by randomizing the scale θ§gt;0 of an inverse Weibull-distributed random variable K , using a PTG-distributed random variable Θ with the same power parameter p>0 .

### 6.2. New relationship between logistic and Gumbel distributions

Appendix B gives an alternative proof of a well-known result in distribution theory [13]: the difference between two independent and identically distributed (i.i.d.) standard Gumbel random variables has the standard logistic law.

By this fact, and with the results from the last section, we introduce a new relationship between a mean-zero logistically distributed random variable and a mean-zero Gumbel-distributed random variable with the same scale parameter.

When a random variable X is mean-zero Gumbel-distributed with CDF given in equation (4.8), then we have from the GMP in equation (4.10) that


(6.13) $$\mathbb{P}\,\left\{X\leq x_{1}{⊕}_{-b}x_{2}|X\leq x_{1}\right\}=\mathbb{P}\,\left\{X\leq x_{2}\right\},$$

where from equation (5.10), for $x_{1},x_{2}\in \left(-\infty ,\infty \right]$ and b>0 , one has


(6.14) $$x_{1}{⊕}_{-b}x_{2}=E[x_{1}\wedge (x_{2}+bZ)],$$

with Z a standard logistically distributed random variable, whose CDF is given in equation (5.11).

We are thus connecting the mean-zero logistically distributed random variable b⁢Z to the mean-zero Gumbel-distributed random variable X , via equations (6.13) and (6.14) and 6_14 rather than by taking differences of i.i.d. copies of X .

## 7. Conclusions and future research

We showed that, when the generator of arithmetic is a strictly monotonic map of all of [0,∞) onto the support [ℓ,u] of a random variable X , then the law of X satisfies a generalization of the memoryless property, in which ordinary addition is replaced by the strictly increasing binary operation ${⊕}_{G}$ in the monoid $\left(\left[\ell ,u\right]:{⊕}_{G},G\,\left(0\right)\right)$ . We illustrated the result with two examples, for which the induced pseudo-sums also have a direct probabilistic and financial interpretation as an arbitrage-free contingent-claim value. In both cases, we are then able to connect two distinct probability distributions in a novel way.

In our view, future research should extend the use of pseudo-analytic arguments in pricing and financial modelling, as well as in probability and statistics.

For instance, a further use of pseudo-analysis, with great potential in both financial mathematics and probability, is linked to the observations below, following straight from insights in Aczél [1], Castagnoli [3] and Pap [6].

Let us first give a definition.

**Definition 7.1** ( G -normality). Let G be a generator satisfying the requirements in §2, and $G^{-1}$ its inverse. A random variable Y is said to be G -normal, if $G^{-1}\,\left(Y\right)$ is normally distributed, so that its probability density function is


(7.1) $$\frac{1}{\sigma \sqrt{2\pi }}e^{-\frac{1}{2}{\left(\frac{G^{-1}(y)-\mu }{\sigma }\right)}^{2}}\frac{\text{d}}{\text{d}y}G^{-1}(y).$$

In other words, if Φ(x;μ,σ) indicates CDF of a normal random variable with mean μ and standard deviation σ , then Y is G -normal if its CDF is $\Phi \,\left(G^{-1}\,\left(y\right);\mu ,\sigma \right)$ , and the density (probability density function, PDF) is the one given in equation (7.1).

An immediate example is represented by the lognormal random variable, which one obtains by exponentiating a normal random variable [26]. In fact, if X is normal, then $Y=G(X)=e^{X}$ is clearly lognormal. However, this implies that $G^{-1}(y)=\log(y)$ and that the CDF of Y is Φ(log⁡(y);μ,σ) , with the corresponding PDF:


$$\frac{1}{y\sigma \sqrt{2\pi }}e^{-\frac{1}{2}{\left(\frac{\log(y)-\mu }{\sigma }\right)}^{2}},$$

as expected.

Now, let $X_{1},X_{2},...,X_{n}$ be a collection of i.i.d. random variables and define the *pseudo-partial-sum*:


$$S_{n}=X_{1}{⊕}_{G}X_{2}{⊕}_{G}...{⊕}_{G}X_{n}=G\left(\sum_{i=1}^{n}G^{-1}(X_{i})\right).$$

Then, for n→∞ , the G -standardized quantity


(7.2) $$G\left(\frac{G^{-1}(S_{n})-E[G^{-1}(S_{n})]}{\sigma [G^{-1}(S_{n})]}\right)$$

will converge to a standard G -normal random variable with CDF $\Phi (G^{-1}(x);0,1)$ , where $\sigma [G^{-1}(Y_{n})]$ is the finite standard deviation of $G^{-1}(S_{n})=\sum_{i=1}^{n}G^{-1}(X_{i})$ .

If the i.i.d. random variables $X_{1},X_{2},...,X_{n}$ are positive and their density function is square-integrable, and we choose $G(X)=e^{X}$ , we can immediately recognize the so-called Gibrat’s law [27], in the convergence of equation (7.2) to its lognormal limit.

A natural question is therefore to ask what do we get for different generators; and a very interesting case emerges for G(X)=log⁡(X) . In fact, we immediately get that


$$S_{n}=X_{1}{⊕}_{G}X_{2}{⊕}_{G}\ldots {⊕}_{G}X_{n}=\log\left(e^{X_{1}}+e^{X_{2}}+\cdots +e^{X_{n}}\right),$$

where $\log\left(\sum_{i=1}^{n}e^{x_{i}}\right)$ is the *LogSumExp* function $LSE(x_{1},...,x_{n})$ commonly used in machine learning as a smooth approximation to the maximum function $\max(x_{1},...,x_{n})$ [16].

If, again, we assume that the random variables $X_{1},X_{2},...,X_{n}$ are positive, i.i.d., and with a square-integrable density, then the limit law of the pseudo-partial-sum $S_{n}$ strongly recalls a Gumbel (Type I) distribution [13], suggesting a non-trivial but yet-to-be-investigated connection with fundamental results in extreme value theory, for example, the Fisher–Tippett–Gnedenko theorem [25].

The alleged connection strengthens, if we look back at the distributions of §6, and recognize both the Gumbel and the Weibull as two of the three extreme value distributions, and we see that both are linked to the GMP. Curiously, the only extreme value distribution missing in the list seems to be the Fréchet but also here we can notice a simple fact. As we wrote in §4, the Pareto random variable is the variable showing the GMP when x⊕y=x×y , and the Pareto is a fat-tailed random variable linked to the Fréchet [25]. All in all, it seems that there are still a large amount of things to be clarified and possibly discovered using pseudo-sums as we suggested.

## Data Availability

This article has no additional data.

## Footnotes

^1^
Naturally, since the random variable *X* is continuous, our notation for the support of its law also captures sets of the form (ℓ,h],[ℓ,h) , and (ℓ,h) , where ` can be −∞ and *h* can be +∞.

^2^
To interpret the implications of the restriction in equation (2.5), we first observe that if the generator *G* is strictly monotonic, then so is its inverse *G* ^−1^. Furthermore, in our context, demanding associativity of a strictly increasing binary operation converts a strictly increasing map from *A* × *A* to *A*—namely, *PS*(*x*_1_, *x*_2_) ≡ *x*_1^ $\hat{⊕}$^_ *Gx*_2_, with *x*_1_, *x*_2_ ϵ *A*—into a strictly monotonic map from *A* to R , namely, *$G^{-1}\left(x\right),x\;\epsilon \;A$ *.

## Appendix A. More on the conjugate power Dagum random variable

The CDF of a two-parameter CPD random variable $D_{S,b}$ is given by

(A 1)
$$F_{D_{S,b}}(d;b,S)\equiv {\left(1+{\left(\frac{d}{S}\right)}^{-\frac{1}{b}}\right)}^{b-1},\;d,S>0,b\in (0,1).$$

We refer to S>0 as the scale parameter in the law of $D_{S,b}$ .

The PDF of $D_{S,b}$ is then equal to

(A 2)
$$f_{D_{S,b}}(d;b,S)=\frac{1-b}{Sb}{\left(1+{\left(\frac{d}{S}\right)}^{-\frac{1}{b}}\right)}^{b-2}{\left(\frac{d}{S}\right)}^{-\frac{1}{b}-1},\;d>0,b\in (0,1),S>0.$$

As a result, the mean of the two-parameter CPD random variable X is given by

(A 3)
$$\begin{array}{ll} E[D_{S,b}] & =\int_{0}^{\infty }d\frac{1-b}{Sb}{\left(1+{\left(\frac{d}{S}\right)}^{-\frac{1}{b}}\right)}^{b-2}{\left(\frac{d}{S}\right)}^{-\frac{1}{b}-1}\text{d}d\\ & =\frac{1-b}{b}\int_{0}^{\infty }{\left(\frac{d}{S}\right)}^{-\frac{1}{b}}{\left(1+{\left(\frac{d}{S}\right)}^{-\frac{1}{b}}\right)}^{b-2}\text{d}d\\ & =\frac{1-b}{b}\int_{0}^{\infty }\frac{{\left(\frac{d}{S}\right)}^{-\frac{1}{b}}}{1+{\left(\frac{d}{S}\right)}^{-\frac{1}{b}}}{\left(1+{\left(\frac{d}{S}\right)}^{-\frac{1}{b}}\right)}^{b-1}\text{d}d \end{array},$$

for b∈(0,1),S>0 . Let

(A 4)
$$\pi =\frac{{\left(\frac{d}{S}\right)}^{-\frac{1}{b}}}{1+{\left(\frac{d}{S}\right)}^{-\frac{1}{b}}}={\left(1+{\left(\frac{d}{S}\right)}^{\frac{1}{b}}\right)}^{-1}$$

be a change of integrating variable. Then,

(A 5)
$${\left(\frac{d}{S}\right)}^{\frac{1}{b}}=\frac{1}{\pi }-1=\frac{1-\pi }{\pi }.$$

Reciprocating both sides of equation (A 5), one gets

$${\left(\frac{d}{S}\right)}^{-\frac{1}{b}}=\frac{\pi }{1-\pi }.$$

Now let us add one on both sides,

(A 6)
$$1+{\left(\frac{d}{S}\right)}^{-\frac{1}{b}}=1+\frac{\pi }{1-\pi }=\frac{1}{1-\pi }.$$

Solving equation (A 5) for *d*, one obtains

(A 7)
$$d=S{\left(\frac{1-\pi }{\pi }\right)}^{b}.$$

Hence,

(A 8)
$$\text{d}d=Sb{\left(\frac{1-\pi }{\pi }\right)}^{b-1}\frac{-1}{\pi^{2}}\text{d}\pi .$$

Under the decreasing change of variable in equation (A 4), the limits of integration change from d=0 and d=∞ to π=1 and π=0 , respectively. Substituting equations (A 4), (A 6) and (A 8) in equation (A 3) implies that, for all b∈(0,1) ,

(A 9)
$$\begin{array}{ll} E[D_{S,b}] & =\frac{1-b}{b}\int_{0}^{1}\pi {\left(\frac{1}{1-\pi }\right)}^{b-1}Sb{\left(\frac{1-\pi }{\pi }\right)}^{b-1}\frac{1}{\pi^{2}}\text{d}\pi \\ & =(1-b)S\int_{0}^{1}\pi^{-b}\text{d}\pi \\ & =S\pi^{1-b}{|}_{\pi =0}^{\pi =1}\\ & =S \end{array}.$$

Therefore, when the CDF of the two-parameter CPD-distributed random variable $D_{S,b}$ is given by equation (A 1), for d>0 , b∈(0,1) , S>0 , then the mean of $D_{S,b}$ is just the scale parameter S>0 .

If S=1 , we denote $D_{1,b}$ by $D_{b}$ , and the CDF simplifies to

(A 10)
$$F_{D_{b}}(d;b,1)\equiv {\left(1+d^{-\frac{1}{b}}\right)}^{b-1},\;d>0,b\in (0,1).$$

By equation (A 10), the mean of $D_{b}>0$ is trivially 1 for all b∈(0,1) .

## Aa. The Lorenz curve and the Gini index of $D_{b}$

It is not hard to see that a random variable $X∼D_{b}$ could represent a valid tool to model phenomena like wealth, income or portfolio losses (when considered as positive quantities, as commonly done in risk management). In other words, the CDF of *X* can be seen as a statistical size distribution, in the definition of [13].

For this reason, we provide two useful quantities related to $D_{b}$ : the associated Lorenz curve and the corresponding Gini index [21].

Let $F_{G}(g)$ , g>0 be the CDF of the positive random variable G . Since F is increasing, it has a well-defined inverse called the quantile function Q(p) , p∈[0,1] . The Lorenz function. L(u) , u∈[0,1] , is defined as the integral of the quantile function Q(p) from p=0 to p=u , divided by the mean of the positive random variable [13]. Whenever the mean of *G* is 1, as for a random variable with CDF equal to $D_{b}$ , we just have the numerator.

Now, given the CDF of the mean-one CPD, as per equation (A 10), the quantile function of a CPD-distributed random variable is

(A 11)
$$Q_{D_{b}}(p)={\left(p^{\frac{1}{b-1}}-1\right)}^{-b},\;p\in (0,1),b\in (0,1).$$

Hence, the related Lorenz curve is equal to

(A 12)
$$\begin{array}{ll} L_{D_{b}}(u) & \equiv \int_{\;\;0}^{u}Q(p)\text{d}p\\ & =\int_{\;\;0}^{u}{\left(p^{\frac{1}{b-1}}-1\right)}^{-b}\text{d}p\\ & ={\left(u^{\frac{1}{b-1}}-1\right)}^{-b}\left(u-u^{\frac{b}{b-1}}+{\left(u^{\frac{1}{b-1}}-1\right)}^{b}\right) \end{array}.$$

The Gini index then reads

(A 13)
$${Gini}_{D_{b}}=\frac{\Gamma (1-b)\Gamma (2-b)}{\Gamma (2-2b)}-1,$$

and it corresponds to twice the area between *u* and $L_{D_{b}}(u)$ for u∈[0,1] .

For more details on the Lorenz curve and the Gini index, we refer to references [13] and [21].

## Appendix B. Difference of two independent and identically distributed standard Gumbel random variables

The standard Gumbel CDF is:

(B 1)
$$F_{X}(x)=e^{-e^{-x}},\;x\in R.$$

Differentiating gives the PDF of a standard Gumbel-distributed random variable, that is:

(B 2)
$$f_{X}(x)=e^{-e^{-x}}e^{-x}=e^{-e^{-x}-x},\;x\in R.$$

The PDF of the difference of 2 i.i.d. standard Gumbel random variables is therefore

(B 3)
$$f_{X-Y}(z)=\int_{-\infty }^{\infty }f_{X}(z+y)f_{Y}(y)\text{d}y,\;z\in R.$$

We are interested in the CDF of the difference, so we integrate on *z* from −∞ up to *k* and use Fubini:

(B 4)
$$F_{X-Y}(k)=\int_{-\infty }^{\infty }\int_{-\infty }^{k}f_{X}(z+y)\text{d}zf_{Y}(y)\text{d}y,\;k\in R.$$

Letting x=z+y be a change of the inner integrating variable *z*, the desired CDF can be written as

(B 5)
$$F_{X-Y}(k)=\int_{-\infty }^{\infty }F_{X}(k+y)f_{Y}(y)\text{d}y,\;k\in R.$$

Substituting equations (B 1) and (B 2) in equation (B 5), one gets

(B 6)
$$F_{X-Y}(k)=\int_{-\infty }^{\infty }e^{-e^{-k-y}}e^{-e^{-y}-y}\text{d}y=\int_{-\infty }^{\infty }e^{-(1+e^{-k})e^{-y}}e^{-y}\text{d}y,\;k\in R.$$

Let $u=e^{-y}$ be a change of integrating variable. Then, y=−log⁡u and $dy=-\frac{du}{u}$ . The integral limits become 0 and ∞ , so the CDF of the difference of 2 i.i.d. standard Gumbel random variables now reads as

(B 7)
$$\begin{array}{ll} F_{X-Y}(k) & =\int_{0}^{\infty }e^{-(1+e^{-k})u}u\frac{\text{d}u}{u}\\ & =-\frac{e^{-(1+e^{-k})u}}{1+e^{-k}}{|}_{u=0}^{u=\infty }\\ & =(1+e^{-k}{)}^{-1},\;k\in R \end{array}.$$

which is the standard logistic CDF.

## Appendix C. Randomizing the scale of an inverse Weibull-distributed random variable using a power-transformed gamma

From the CDF of the PTG-distributed random variable Θ>0 in equation (6.4) , we obtain the following PDF:

(C 1)
$$\begin{array}{ll} f_{\Theta }(\theta ;S,\alpha ,p) & =\frac{1}{\Gamma (\alpha )}{\left({\left(\frac{\theta }{S}\right)}^{p}\right)}^{\alpha -1}e^{-{\left(\frac{\theta }{S}\right)}^{p}}p\frac{\theta^{p-1}}{S^{p}}\\ & =\frac{p\theta^{p\alpha -1}e^{-{\left(\frac{\theta }{S}\right)}^{p}}}{S^{p\alpha }\Gamma (\alpha )},\;\theta >0,S>0,\alpha >0,p>0 \end{array}.$$

Now, consider randomizing the scale parameter θ>0 of an inverse Weibull-distributed random variable *K*, by using an independent PTG-distributed random variable Θ>0 . Let *D* denote the result.

For d>0 , S>0 , α>0 , p>0 , the CDF of D is given by the integral

(C 2)
$$\begin{array}{ll} F_{D}(d;S,p,\alpha ) & =\int_{\;\;0}^{\infty }F_{K}(d;\theta )f_{\Theta }(\theta ;S,\alpha ,p)\text{d}\theta \\ & =\int_{\;\;0}^{\infty }e^{-{\left(\frac{\theta }{d}\right)}^{p}}\frac{p\theta^{p\alpha -1}e^{-{\left(\frac{\theta }{S}\right)}^{p}}}{S^{p\alpha }\Gamma (\alpha )}\text{d}\theta \\ & =\frac{p}{S^{p\alpha }\Gamma (\alpha )}\int_{\;\;0}^{\infty }\theta^{p\alpha -1}e^{-\theta^{p}\left(d^{-p}+S^{-p}\right)}\text{d}\theta \end{array}.$$

Let $g=\theta^{p}\left(d^{-p}+S^{-p}\right)$ be a change of integrating variable. Then, $\theta =g^{\frac{1}{p}}{\left(d^{-p}+S^{-p}\right)}^{-\frac{1}{p}}$ and $\text{d}\theta =\frac{g^{\frac{1}{p}-1}}{p}{\left(d^{-p}+S^{-p}\right)}^{-\frac{1}{p}}$ .

For d>0 , S>0 , α>0 , p>0 , the CDF of D becomes

(C 3)
$$\begin{array}{ll} F_{D}(d;S,p,\alpha ) & =\frac{p}{S^{p\alpha }\Gamma (\alpha )}\int_{\;\;0}^{\infty }\{g^{\frac{1}{p}}{\left(d^{-p}+S^{-p}\right)}^{-\frac{1}{p}}{\}}^{p\alpha -1}e^{-g}\frac{g^{\frac{1}{p}-1}}{p}{\left(d^{-p}+S^{-p}\right)}^{-\frac{1}{p}}\text{d}g\\ & =\frac{S^{-p\alpha }{\left(d^{-p}+S^{-p}\right)}^{-\alpha }}{\Gamma (\alpha )}\int_{\;\;0}^{\infty }g^{\alpha }e^{-g}\text{d}g\\ & ={\left(1+{\left(\frac{d}{S}\right)}^{-p}\right)}^{-\alpha },\;d>0,S>0,p>0,\alpha >0 \end{array}.$$

This is nothing more than the CDF of a Dagum-distributed random variable [13].

We thus conclude that randomizing the scale θ>0 of an inverse Weibull-distributed random variable K>0 , using an independent PTG-distributed random variable Θ>0 , results in a Dagum-distributed random variable.
